# Meta‐Analysis of the Efficacy of Spirulina Intervention in Mitigating the Negative Impact of Heat Stress on Production Physiology and Health Indices of Broilers

**DOI:** 10.1111/jpn.70016

**Published:** 2025-10-08

**Authors:** Christian Anayo Mbajiorgu, Ifeanyichukwu Princewill Ogbuewu, Monnye Mabelebele

**Affiliations:** ^1^ Department of Agriculture and Animal Health University of South Africa Florida South Africa

**Keywords:** antioxidative capacity, blood characteristics, broilers, carcass traits, growth, heat stress, spirulina

## Abstract

There is an increasing number of published studies on the effect of spirulina (an aquatic plant known for its high nutritional value and potential health benefits) intervention on productivity and health of heat‐stressed broilers. However, the effect of spirulina intervention on the performance of broilers exposed to heat stress is poorly understood. A better understanding of the productivity of heat‐stressed broilers on spirulina intervention will assist in utilizing these data in decision‐support systems in the poultry industry. Therefore, this study aimed to determine the effectiveness of spirulina intervention in enhancing production physiology and health indices of heat‐stressed broilers using a meta‐analysis approach. A detailed search performed on PubMed, Embase, Google Scholar, ScienceDirect, Scopus, and Web of Science databases on the topic identified 865 publications following the Preferred Reporting Items for Systematic Reviews and Meta‐analyses (PRISMA) guidelines. Thirteen peer‐reviewed studies comprising 4904 broilers exposed to heat stress conditions were used for meta‐analysis. Raw mean differences (RMD) between the heat‐stressed broilers with and without spirulina intervention were used to calculate the effect sizes. Heat‐stressed broilers on spirulina intervention had their average daily feed intake (ADFI), feed conversion ratio (FCR), and average daily gain (ADG) enhanced by 3.39 g/bird/day (*p* = 0.002), −0.08 (*p* = 0.010), and 2.83 g/bird/day (*p* < 0.001), respectively when compared to those in control group. Restricted subgroup analysis showed that moderators (broiler strains, dose level of spirulina, and production phases) affected ADFI, FCR, and ADG in heat‐stressed broilers on spirulina intervention. Dressing percentage (RMD = 1.60%; *p* < 0.001), and weights of breast, thigh, liver, heart, gizzard, spleen, and thymus were higher, but the abdominal fat weight was lower in response to spirulina intervention. Additionally, spirulina intervention increased the levels of hemoglobin (Hb), red blood cell (RBC), white blood (WBC), total protein, albumin, and globulin, and decreased the levels of uric acid, creatinine, total cholesterol, triglycerides, low‐density lipoprotein (LDL), alanine aminotransferase (ALT), and aspartate aminotransferase (AST) in broilers exposed to heat stress conditions. The results indicate significant increase in superoxide dismutase (SOD) and glutathione peroxidase (GPx), immunoglobulin G (IgG), immunoglobulin M (IgM), immunoglobulin A (IgA), and reduction in malondialdehyde (MDA) in broilers in comparison with controls. It can be concluded that spirulina intervention has the potential to improve growth performance, organ and carcass parameters, blood characteristics, immune functions, and antioxidative capacity of broilers exposed to heat‐stress. These findings can be used by farmers, feed manufacturers, poultry nutritionists, and policymakers in decision‐support systems to advance the use of spirulina in the poultry industry.

## 1 Introduction

The poultry industry contributes significantly to global food production, with broiler meat being one of the most consumed meat sources globally. However, one major challenge confronting the broiler industry, especially in tropical and subtropical countries is high ambient temperature (Awad et al. 2020; Moustafa et al. 2021). Apalowo et al. (2024) found that broilers performed optimally when the ambient temperature ranged from 21°C to 25°C, however, heat stress may arise when the mean ambient temperature exceeds 30°C. Broilers are prone to heat stress because of their high metabolic rate, which produces more body heat, resulting in high mortality rates (Abo Ghanima et al. 2020; He et al. 2020). Exposure to heat stress has been linked to reduced broiler growth, immune function, imbalance redox status, and blood metabolite disorder, leading to significant economic losses for the poultry industry (He et al. 2020; Moustafa et al. 2021; Attia et al. 2023; Chaudhary et al. 2023). Furthermore, heat stress increases reactive oxygen species (ROS) and oxidative damage, leading to immune cell apoptosis and intestinal barrier dysfunction (Habashy et al. 2019), resulting in the passage of harmful substances into the body system (Koch et al. 2019; Hirakawa et al. 2020).

As a result, creating solutions to mitigate the negative impact of heat stress on broiler performance is crucial for broiler welfare, while ensuring the economic sustainability of the broiler industry. The use of feed supplements such as trace elements, vitamins, prebiotics, and probiotics as a nutritional strategy for mitigating the negative impact of heat stress on broiler performance has been researched with variable findings (Abdel‐Moneim et al. 2020; Ibrahim et al. 2020; Chaudhary et al. 2023). In recent years, there has been growing interest in using marine plants to enhance chicken performance and health (Basiouni et al. 2022; Shehata et al. 2022). The use of spirulina (*Spirulina platensis*), one such marine plant to mitigate the negative impacts of heat stress in the poultry industry, has been investigated (Hassan et al. 2021; Hassan et al. 2023).

Spirulina is a microalga that grows well in marine and freshwater environments. Spirulina is high in nutrients (protein, lipids, fatty acids, minerals, phytopigments, and vitamins) and bioactive phytochemical compounds such as flavonoids, phenolic acid, phycocyanins, and triterpenoids (Abdel‐Daim et al. 2015; Farag et al. 2016; Abdel‐Moneim et al. 2022a) demonstrated to have several nutritional and medicinal values (El‐Bahr et al. 2020; Attia et al. 2023; Mullenix et al. 2021; Abdelfatah et al. 2024). Moreso, spirulina is a sustainable and renewable feed supplement source, requiring minimal land, water, and energy for production. The nutritional and medicinal benefits of spirulina may be responsible for its diverse pharmacological effects, including antioxidants, immunomodulatory, antimicrobial, antiviral, hepatoprotective, and anti‐inflammatory activities (Farag et al. 2016; Abdel‐Moneim et al. 2022a). Spirulina's antioxidant activity can help reduce oxidative stress and inflammation caused by heat stress, thus enhancing broiler productivity and welfare (Abdel‐Moneim et al. 2020; Hassan et al. 2021) leading to increased profitability.

Over the past years, the effects of spirulina on broiler performance have been inconsistent (Attia et al. 2023; Moustafa et al. 2021; Kolluri et al. 2022; Chaudhary et al. 2023; Hassan et al. 2023), making it difficult to use this information in the decision‐support systems in the poultry industry. These consistent findings on the influence of spirulina intervention on heat‐stressed broilers could be attributed to factors such as diet composition, the quantity of spirulina added to the feed, variable study designs, and several other factors that may affect broiler performance (Ogbuewu et al. 2022). The use of meta‐analysis, a statistical technique that quantitatively aggregates data from multiple studies addressing the same research objectives to resolve conflicting findings and provides a more precise estimate of the intervention in animal agriculture has been reported in broilers (Rusli et al. 2022), laying hens (Ogbuewu et al. 2021), and ruminant (Xin et al. 2025).

Despite the reported ability of meta‐analysis to resolve inconsistent findings and provide a more precise estimate of an intervention, published data on meta‐analysis of spirulina intervention on performance of broilers exposed to heat stress conditions are very scarce and are needed. Thus, the objective of the current study was to determine the efficacy of spirulina intervention in enhancing production physiology and health indices of broilers exposed to heat‐stress conditions using a meta‐analysis approach.

## 2 Materials and Methods

### 2.1 Search Strategy

PubMed, Embase, Web of Science, Scopus, ScienceDirect, and Google Scholar databases were searched between December 2024 and February 2025 for articles that explored the effect of spirulina intervention on production performance and health status of heat‐stressed broilers based on the PRISMA checklist (Page et al. 2021). The reference list of retrieved publications was also searched to identify additional relevant publications. The search keywords were *Spirulina platensis*, spirulina, broilers, broiler chickens, heat stress, thermal stress, performance, productivity, organ weights, carcass, carcass composition, meat quality, blood characteristics, immune responses, and antioxidative capacity using the wildcard (* or $), proximity searching (“…” or “…”), Boolean logic (AND/OR), and alternate spelling (?).

### 2.2 Study Screening and Selection

Research question was framed based on the PICO template, where population (P) is heat‐stressed broilers, intervention (I) is spirulina, Comparators (C) is heat stressed broilers not in spirulina intervention, and outcomes (O) were growth performance (ADFI, FCR, and ADG), carcass traits (dressing percentage, abdominal fat, breast, and thigh), organ weights (liver, heart, gizzard, spleen, thymus, and Bursa of Fabricius), blood [Hb, packed cell volume (PCV), RBC, WBC, total protein, albumin, globulin, uric acid, glucose, creatinine, total cholesterol, triglycerides, high‐density lipoprotein (HDL), LDL, ALT, AST, and alkaline phosphatase (ALP), immunoglobulin (IgG, IgM, and IgA), and antioxidative enzyme capacity (MDA, SOD, and GPx)]. Studies were included based on the following criteria (i) randomized and controlled studies; (ii) studies provided sufficient data for calculation of effect sizes; (iii) studies offered diets and water free from antibiotics or other growth promoters; and (iv) trials reported the mean of the control and treatment groups of measured outcomes. Trials were excluded if they (i) were not performed in heat‐stressed broilers; (ii) did not measure outcomes of interest; and (iii) blended spirulina with other growth promoters.

**Figure 1:**
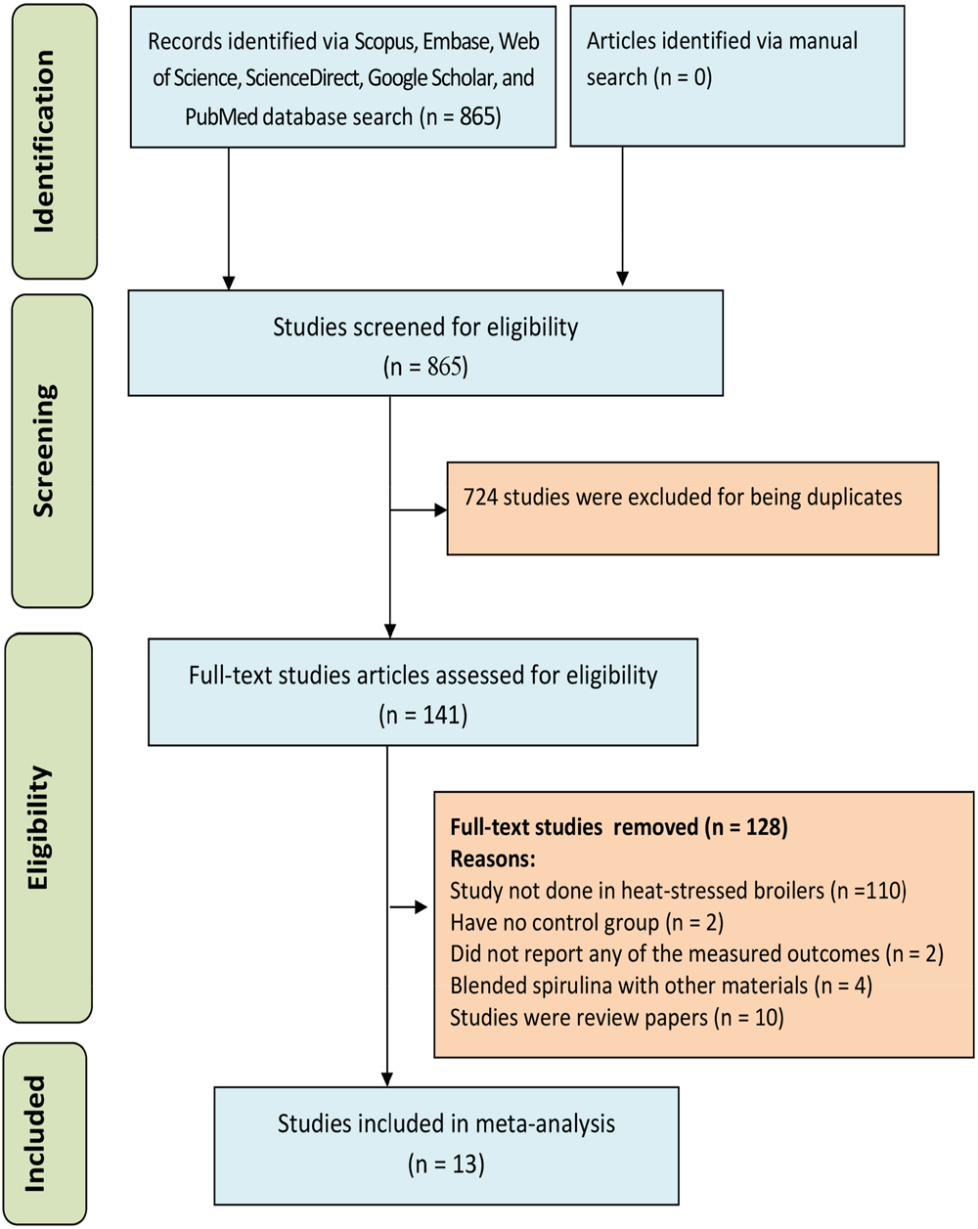
The study selection flow chart of the meta‐analysis. [Color figure can be viewed at wileyonlinelibrary.com]

The results of the searches were retrieved and imported into Zotero (Version 7.0), and duplicate studies emerged. A 2‐step study selection method was adopted, as described by Orzuna‐Orzuna et al. (2022) and Ogbuewu et al. (2025). First, the titles and abstracts of the selected studies were independently screened to exclude reviews and trials were not in heat‐stressed broilers. Second, the full‐text studies that passed the title and abstract screening were reviewed based on the pre‐defined inclusion and exclusion criteria to identify relevant publications to include in the meta‐analysis. Figure 1 revealed that 13 publications out of 865 identified publications met the inclusion criteria.

### 2.3 Data Extraction and Statistical Analysis

Data was extracted from the 13 peer‐reviewed articles that met the inclusion criteria using Microsoft Excel (Windows 10.0 version). The extracted information includes the first author's surname, publication year, study country/continent, number of broilers included in the treatment and control groups, and moderators. The moderators considered in this study to explain the heterogeneity variance were (i) broiler strains (Cobb, Ross, and CARIBROvisha); (ii) dose level of spirulina (0.1–3.0%: ≤ 1.0% and > 1.0%); and (iii) production phases [starter phase (1–21 days), finisher phase (22–42 days), and overall phase (1–42 days)]. Studies that presented results in graphical format were digitized using the WebplotDigitizer Version 4.5 (Rohatgi 2022). Effect sizes across studies were combined using a random‐effects model and results were presented as RMD at a 95% confidence interval (CI) for each of the included study. Heterogeneity was calculated using the Inconsistency index (*I* ^2^) (Higgins 2003). Meta‐regression analysis was done for measured outcomes with ten or more studies because of high statistical power (Hernández‐García et al. 2024). The restricted subgroup effect was not assessed in strata with < 3 comparisons because of low statistical power (Ogbuewu et al. 2020). The subgroup effect of CARIBROvisha on growth performance was not evaluated because of the low sample size. Publication bias was assessed statistically using Egger's regression test (Egger et al. 1997). Furthermore, sensitivity analyses were performed to determine the reliability of pooled results. The statistical model used for the meta‐regression analysis was $Y_{ij}=\beta_{0}+\beta_{1}X_{ij}+e_{ij},$ where *Y*_ij_ = Dependent variable, *β*_0_ = constant/intercept, *β*_1_ = regression coefficient, and *X*_ij_ = moderators, *e*_i_j = residual error. All the analyses were done using OpenMEE software (Wallace et al. 2016). Results were deemed significant at a 5% probability level.

## 3 Results

The article selection process, as presented in Figure 1, showed that the search yielded 865 trials, of which 13 articles passed the inclusion criteria. The description of the published articles included in the meta‐analysis is summarized in Table 1. The studies were published between 2018 and 2024. Four thousand nine hundred and four (4904) broilers with 928 and 3976 broilers for the control and experimental groups, respectively. Most studies were published in Egypt (46%), Iran (23%), Saudi Arabia (15%), India (8%), and the USA (8%). Ross, Cobb, and CARIBROvisha were the strains used, with Ross being the most prominent. The majority of the studies were conducted in Africa and Asia. Production phases were the starter phase (1–21 days), finisher phase (22–42 days), and overall phase (1–42 days), while the dose levels of spirulina ranged from 0.1% to 3.0%.

**Table 1:** Description of studies utilized for meta‐analysis.

Studies	Country	Continent	Number of broilers used		Moderator variables		
			CG	EG	DL	BS	PP
Mirzaie et al. (2018)	Iran	Asia	50	200	0.5, 1, 2	Cobb	22–42
Moustafa et al. (2021)	Egypt	Africa	60	240	0.5, 1, 1.5	Cobb	22–42
Abdel‐Moneim et al. (2022a)	Egypt	Africa	50	400	0.5, 1	Ross	^b^
Abdel‐Moneim et al. (2022b)	Egypt	Africa	50	400	0.5, 1	Ross	1.0–42
Elbaz et al. (2022)	Egypt	Africa	120	480	0.1, 0.2, 1, 2	Ross	^b^
Kolluri et al. (2022)	India	Asia	72	288	0.5, 1, 1.5	a	1.0–42
Shafer (2022)	Saudi Arabia	Asia	100	300	0.5, 1, 1.5	Ross	1.0–21
Abed et al. (2023)	Egypt	Africa	50	100	0.1, 0.2	Ross	1.0–42
Attia et al. (2023)	Saudi Arabia	Asia	120	480	0.1	Ross	22–42
Chaudhary et al. (2023)	USA	North America	36	108	3	Cobb	1.0–42
Hassan et al. (2023)	Egypt	Africa	120	480	0.1	Ross	22–42
Nazmi et al. (2024)	Iran	Asia	60	300	0.25, 0.5, 0.75, 1	Ross	1.0–42
Ranjbarinasab et al. (2024)	Iran	Asia	40	200	1	Ross	1.0–42

Abbreviations: BS, broiler strains used; CG, control group; DL, dose level of spirulina added to the diet in percentage (%); EG, experimental group; PP, production phases in days.

a CARIBROvisha.

b 1.0–21, 22–42, 1–42.

### 3.1 Growth Performance

Results show significantly high ADFI (RMD = 3.39 g/b/d; *p* = 0.002) and ADG (RMD = 2.82 g/bird/day; *p* < 0.001), and low FCR (RMD = −0.08; *p* = 0.010) in heat‐stressed broilers on spirulina treatment compared to control treatment with evidence large heterogeneity (*I* ^2^ = 96–98%; *p* < 0.001) as shown in Table 2. The Egger regression test, which is not significant. The subgroup effects of moderators on growth variables of heat‐stressed broilers are presented in Table 3. Heat‐stressed broilers on ≤ 1.0% spirulina intervention had better ADFI (RMD = 3.91 g/bird/day; *p* < 0.001), FCR (RMD = −0.08; *p* < 0.001), and ADG (RMD = 2.92 g/bird/day; *p* < 0.001) than broilers in the control groups. Likewise, heat‐stressed broilers on > 1.0% spirulina intervention recorded higher ADG (RMD = 2.48 g/b/d; *p* < 0.001) than broilers in the control groups. Heat‐stressed broilers on ≤ 1.0% spirulina intervention had better FCR than those on > 1.0% spirulina intervention. In comparison with the control, spirulina treatment improved ADFI, FCR, and ADG in the Cobb and Ross strains, except for FCR, which was not improved in the Cobb strain. Subgroup analysis results show that ADFI of heat‐stressed broilers on spirulina intervention during the starter production phase (1–21 days) was increased by 11.50 g/bird/day (*p* < 0.001) compared to the finisher production phase (22–42 days), and overall production phase (1–42 days). Results showed that FCR improved in heat‐stressed broilers during the starter and finisher production phases. In addition, ADG values of heat‐stressed broilers on spirulina intervention during the starter, finisher, and overall production phases were significantly higher than those in the control groups. Meta‐regression results as presented in Table 4 found no evidence of significant relationships between growth performance traits and selected moderator variables.

**Table 2:** Growth performance of heat‐stressed broilers on spirulina intervention.

Parameters	Random‐effects model				Heterogeneity		ET
	*n*	RMD	95% CI	*p* value	*I* ^2^ %	*p* value	*p* value
ADFI (g/day/bird)	42	3.39	1.23, 5.55	0.002	97	< 0.001	0.106
FCR	41	−0.08	−0.14, −0.02	0.010	98	< 0.001	0.410
ADG (g/day/bird)	41	2.82	1.84, 3.80	< 0.001	96	< 0.001	0.091

Abbreviations: ADFI, average daily feed intake; ADG, average daily gain; CI, confidence interval; ET, Egger's test; FCR, feed conversion ratio; *I* ^2^, inconsistency index; n, number of comparisons; RMD, raw mean difference.

**Table 3:** Impact of moderators on growth metrics of heat‐stressed broilers on spirulina intervention.

Traits	Moderators	RMD	95% CI	*p* value	Heterogeneity	
					*I* ^2^ (%)	*p* value
ADFI (g/bird/day)	Dose level (%)					
	≤ 1.0	3.91	1.20, 6.62	0.005	97	< 0.001
	> 1.0	1.63	−1.11, 4.36	0.243	89	< 0.001
	Broiler strains					
	Ross	3.91	1.21, 6.61	0.004	97	< 0.001
	Cobb	3.99	0.74, 7.24	0.016	78	< 0.001
	PP (days)					
	1–21	11.50	6.26, 16.75	< 0.001	98	< 0.001
	22–42	0.29	−1.46, 2.04	0.748	61	0.002
	1–42	1.89	−1.47, 5.24	0.270	96	< 0.001
FCR	Dose level (%)					
	≤ 1.0	−0.08	−0.13, −0.03	0.004	97	< 0.001
	> 1.0	−0.07	−0.27, 0.13	0.506	99	< 0.001
	Broiler strains					
	Ross	−0.10	−0.14, −0.06	< 0.001	95	< 0.001
	Cobb	0.06	−0.36, 0.47	0.792	91	< 0.001
	PP (days)					
	1–21	−0.17	−0.28, −0.06	0.003	99	< 0.001
	22–42	−0.02	−0.20, 0.16	0.829	98	< 0.001
	1–42	−0.07	−0.08, −0.05	< 0.001	7	0.376
ADG (g/bird/day)	Dose level (%)					
	≤ 1.0	2.92	1.77, 4.06	< 0.001	96	< 0.001
	> 1.0	2.48	0.44, 4.51	0.017	96	< 0.001
	Broiler strains					
	Ross	2.60	1.48, 3.72	< 0.001	97	< 0.001
	Cobb	5.03	1.26, 8.79	0.009	96	< 0.001
	PP (days)					
	1–21	1.68	0.31, 3.05	0.016	96	< 0.001
	22–42	3.47	1.04, 5.91	0.005	96	< 0.001
	1–42	2.98	1.05, 4.91	0.002	96	< 0.001

Abbreviations: ADFI, average daily feed intake; ADG, average daily gain; FCR, feed conversion ratio; PP, production phases.

**Table 4:** Relationships between moderators and growth performance data in heat‐stressed broilers.

Growth traits	Moderators	*Q*_B_	*Q*_M_	*p* value	*R* ^2^ (%)
ADFI	Dosel level	4.08	0.23	0.629	0
	Broiler strains	4.12	0.54	0.765	0
	PP	12.17	5.67	0.059	8
FCR	Dosel level	−0.08	0.03	0.874	0
	Broiler strains	−0.11	3.03	0.220	3
	PP	−0.17	2.66	0.265	2
ADG	Dosel level	2.923	0.09	0.766	0
	Broiler strains	2.607	3.66	0.161	4
	PP	1.65	1.22	0.543	0

Abbreviations: PP, production phases; Q_B_, regression coefficient (or beta); Q_M_, coefficient of moderators; R^2^, amount of heterogeneity explained by the covariates.

### 3.2 Carcass and Internal Organ Characteristics

Table 5 revealed that spirulina treatment increased the dressing percentage, weights of breast, thigh, and reduced the abdominal fat content by 1.60% (*p* < 0.001), 1.75% (*p* < 0.001), 0.44% (*p* = 0.039), and −0.31% (*p* = 0.018), respectively, in heat‐stressed broilers compared to the control treatment. Results also indicate that the weights of liver (RMD = 0.24%; *p* = 0.036), heart (RMD = 0.05%; *p* < 0.001), and gizzards (RMD = 0.13%; *p* = 0.004) were increased by spirulina treatment. Spleen and thymus weighed increased by 0.03% (*p* = 0.042) and 0.02% (*p* = 0.011), respectively, in response to spirulina intervention. In contrast, Bursa of Fabricius was not significantly different from controls. The Egger's test results show no evidence of significant publication bias.

**Table 5:** Carcass and organ weight characteristics of heat‐stressed broilers on spirulina intervention.

Parameters (%)	Random‐effects model				Heterogeneity		ET
	*n*	RMD	95% CI	*p* value	*I* ^2^ %	*p* value	*p* value
Dressing percentage	25	1.60	0.91, 2.29	< 0.001	55	< 0.001	0.340
Breast	18	1.75	1.27, 2.22	< 0.001	41	0.109	0.081
Thigh	18	0.44	0.07, 0.81	0.039	31	0.178	0.403
Abdominal fat	18	0.31	−0.06, −0.04	0.018	82	< 0.001	0.084
Liver	16	0.24	0.02, 0.47	0.036	90	< 0.001	0.056
Heart	21	0.05	0.02, 0.08	< 0.001	85	< 0.001	0.262
Gizzard	21	0.13	0.04, 0.22	0.004	88	< 0.001	0.061
Spleen	18	0.03	0.01, 0.05	0.042	77	< 0.001	0.884
Thymus	17	0.02	0.04, 0.03	0.011	87	< 0.001	0.079
Bursa of Fabricius	18	−0.01	−0.02, 0.03	0.147	64	< 0.001	0.390

Abbreviations: CI, confidence interval; ET, Egger's test; *I* ^2^, Inconsistency index; n, number of comparisons; RMD, raw mean difference.

### 3.3 Hemato‐Biochemical Characteristics

The analysis as shown in Table 6 revealed that Hb (*p* < 0.001), RBC (*p* = 0.037), and WBC (*p* = 0.035) increased by 1.27 g/dl, 0.48 × 10^6^/mm^3^, and 3.09 × 10^6^/mm^3^, respectively response to spirulina intervention. In contrast, spirulina intervention did not affect PCV, albumin, glucose, HDL, and ALP in heat‐stressed broilers. Results showed that spirulina increased serum concentrations of total protein and globulin in heat‐stressed broilers. On the other hand, spirulina decreased the serum concentrations of uric acid, creatinine, total cholesterol, triglycerides, LDL, ALT, and AST in heat‐stressed broilers. Egger's regression test results indicate the absence of significant publication bias.

**Table 6:** Hemato‐biochemical values of heat‐stressed broilers on spirulina intervention.

Parameters	Random‐effects model				Heterogeneity		ET
	*n*	RMD	95% CI	*p* value	*I* ^2^ %	*p* value	*p* value
Hemoglobin (g/dl)	17	1.27	0.85, 1.68	< 0.001	91	< 0.001	0.480
PCV (%)	16	1.16	−0.59, 2.91	0.194	95	< 0.001	0.181
RBC (×10^6^/mm^3^)	18	0.48	0.03, 0.93	0.037	93	< 0.001	0.241
WBC (×10^3^/mm^3^)	15	30.09	2.19, 57.98	0.035	90	< 0.001	0.804
Total protein (g/dl)	16	0.43	0.16, 0.71	0.002	98	< 0.001	0.432
Albumin (g/dl)	12	0.09	−0.06, 0.25	0.229	94	< 0.001	0.880
Globulin (g/dl)	12	0.51	0.22, 0.79	< 0.001	93	< 0.001	0.920
Uric acid (mg/dl)	16	−0.74	−1.10, −0.38	< 0.001	92	< 0.001	0.662
Glucose (mg/dl)	13	−13.27	−27.53, 0.98	0.068	99	< 0.001	0.094
Creatinine (mg/dl)	12	−0.11	−0.18, −0.04	0.002	98	< 0.001	0.074
Total cholesterol (mg/dl)	21	−33.22	−42.09, −24.34	< 0.001	97	< 0.001	0.064
Triglycerides (mg/dl)	21	−23.03	−29.99, −16.08	< 0.001	97	< 0.001	0.109
HDL (mg/dl)	18	−1.06	−4.14, 2.01	0.499	98	< 0.001	0.454
LDL (mg/dl)	15	−29.41	−40.12, −18.67	< 0.001	96	< 0.001	0.680
ALT (IU/L)	18	−5.42	−8.32, −2.52	< 0.001	97	< 0.001	0.104
AST (IU/L)	18	−5.07	−8.23, −1.91	0.002	92	< 0.001	0.201
ALP (IU/L)	12	8.39	−3.98, 20.76	0.184	98	< 0.001	0.508

Abbreviations: ALP, alkaline phosphatase; ALT, alanine aminotransferase; AST, aspartate aminotransferase; CI, confidence interval; ET, Egger's test; HDL, high‐density lipoprotein; *I* ^2^, inconsistency index; LDL, low‐density lipoprotein; PCV, packed cell volume; n, number of comparisons; RBC, red blood Cell; RMD, raw mean difference; WBC, white blood cell.

### 3.4 Immune Responses and Antioxidative Enzyme Capacity

Results as presented in Table 7 indicate that serum levels of IgG (RMD = 52.64 mg/dl; *p* < 0.001), IgM (RMD = 66.50 mg/dl; *p* < 0.001), and IgA (RMD = 3.82 mg/dl; *p* < 0.001) increased in response to spirulina intervention. Similarly, spirulina reduced serum concentrations of MDA (RMD = −0.38 nmol/ml; *p* < 0.001) and increased serum levels of SOD (RMD = 5.23 U/ml; *p* < 0.001), and GPx (RMD = 7.07 U/ml; *p* < 0.001) in heat‐stressed broilers as shown in Table 8. Egger's regression test results indicate no evidence of significant publication bias among studies that assessed the impact of spirulina intervention on serum immunoglobulin and antioxidative capacity in heat‐stressed broilers.

**Table 7:** Effect of spirulina intervention on serum immunoglobulin of heat‐stressed broilers.

Parameters	Random‐effects model				Heterogeneity		ET
	*n*	RMD	95% CI	*p* value	*I* ^2^ %	*p* value	*p* value
IgG (mg/dl)	17	3.64	4.82, 6.14	< 0.001	95	< 0.001	0.508
IgM (mg/dl)	17	4.50	6.19, 6.90	< 0.001	95	< 0.001	0.451
IgA (mg/dl)	18	4.33	3.86, 4.51	< 0.001	96	< 0.001	0.976

Abbreviations: CI, confidence interval; ET, Egger's test; *I* ^2^, inconsistency index; IgA, immunoglobulin A; IgG, immunoglobulin G; IgM, immunoglobulin M; n, number of comparisons; RMD, raw mean difference.

**Table 8:** Serum antioxidative capacity of heat‐stressed broilers on spirulina intervention.

Parameters	Random‐effects model				Heterogeneity		ET
	*n*	RMD	95% CI	*p* value	*I* ^2^ %	*p* value	*p* value
MDA (nmol/ml)	15	−0.38	−0.47, −0.30	< 0.001	97	< 0.001	0.802
SOD (U/ml)	14	5.23	4.41, 6.05	< 0.001	95	< 0.001	0.196
GPx (U/ml)	13	7.07	5.78, 8.36	< 0.001	98	< 0.001	0.410

Abbreviations: CI, confidence interval; ET, Egger's test; GPx, glutathione peroxidase; *I* ^2^, inconsistency index; MDA, malondialdehyde; n, number of comparisons; RMD, raw mean difference; SOD, superoxide dismutase.

## 4 Discussion

Although several studies have explored the benefits of spirulina in broiler production, to the best of our knowledge, there is scanty published data on effectiveness of spirulina in improving the performance of heat‐stressed broilers using a meta‐analytical approach. The current study indicated that highest number of studies was undertaken in Egypt (46%) followed by Iran (23%) and Saudi Arabia (15%) possibly due to favorable climate for spirulina growth and supporting government policies on spirulina research and development. The majority of studies used for the current meta‐analysis were published within the last 5 years (2021–2024), which supports the increasing global campaign on the use of algae with proven antioxidant and antimicrobial properties as alternatives to antibiotics growth promoters in animal production (WHO 2024).

Heat stress can lead to oxidative damage, inflammation, and changes in the intestinal microbiota composition in chickens. These alterations can make the chicken more prone to diseases and impair its productivity (Apalowo et al. 2024). Eco‐friendly feed additives are presently being advocated in chicken production because of their several pharmacological activities, including immunostimulatory, antioxidant, anti‐inflammatory, and antimicrobial effects, all of which can assist in improving chicken performance (Shehata et al. 2022). Thus, the present study assessed the effect of spirulina intervention on production traits and health status of heat‐stressed broilers. Pooled analysis results indicated that growth performance data were better for heat‐stressed broilers on spirulina intervention than those in the control group. The poor growth performance observed on the control broilers could be due to a decline in voluntary feed intake, decreased energy availability, and disintegration of the histoarchitecture of the intestine, which are some of the direct negative effects of heat stress on chicken welfare and behavior (Lu et al. 2007). Low ADFI and ADG, as well as increased FCR were reported in heat‐stressed broilers reared from 3 to 5 weeks of age (Awad et al. 2020) support the findings of this meta‐analysis. The poor growth performance of broilers in the control group, also corroborates Song et al. (2018), who showed that heat‐challenged broilers use more energy to adapt to environmental stressors while less energy is allocated to muscle accretion. In contrast, the observed improvement in growth performance of heat‐stressed broilers on spirulina intervention might be due to the ability of antioxidant compounds in spirulina to improve intestinal morphology and absorption capacity (Mendiola et al. 2007; Alwaleed et al. 2021). Other possible mechanisms by which spirulina improves growth performance in heat‐stressed broilers include (i) modulation of intestinal microbiota composition in favor of the growth of beneficial microbes (Alwaleed et al. 2021; Abdelfatah et al. 2024), (ii) direct nutrient effect and improvement in feed digestion (Khan et al. 2020; Alwaleed et al. 2021), (iii) modulation of immune functions (Fathi 2018; Opoola et al. 2019), and (iv) increased antioxidantive capacity due to its richness in antioxidant components, such as ß‐carotene, selenium, astaxanthin, phenolic acid, phycocyanin, vitamin C and E (Park et al. 2018; El‐Hady et al. 2022).

The higher ADFI recorded at ≤ 1.0% spirulina in Ross and Cobb strains during the starter production phase suggests that ≤ 1.0% spirulina could be the dose level that optimizes ADFI during this growth phase. However, these results should be interpreted cautiously due to the wide confidence interval in these subgroups, which may indicate uncertainty or variability in the estimates. Likewise, heat‐stressed Ross strain offered ≤ 1.0% spirulina during the starter and overall production phases had superior FCR than those given > 1.0% spirulina, indicating that ≤ 1.0% spirulina could be the level that optimized FCR in Ross during the starter and overall production phases. In contrast, Ross and Cobb strains that received 1.0% and > 1.0% spirulina in the three production phases gained more weight than the control broilers. These results imply that dose levels of spirulina that optimize growth variables in heat‐stressed broilers are dynamic and dependent on growth performance parameters of interest. However, caution is needed when interpreting these results due to the large heterogeneity between studies, which may impact the overall reliability of the findings. Further studies should be geared toward determining the optimal doses of spirulina that optimize growth in broilers to avoid wastage of spirulina supplements. Meta‐regression results indicate that studied moderators (dose levels, broiler strains, and production phase) were not significantly associated with growth variables in heat‐stressed broilers on spirulina intervention. This suggests that selected moderator variables were not the drivers of inconsistent findings among studies that assessed the impact of spirulina on the growth performance in heat‐stressed broilers. This also implies the low predictive power of dose level, broiler strains, and production phase as modifiers of ADFI, FCR, and ADG in heat‐stressed broilers. The lack of significant associations between the studied moderators and growth performance metrics might be attributed to limited statistical power, resulting in small subgroup sizes.

Carcass traits and meat quality in broilers are affected by exposure to heat stress (Awad et al. 2020; Gonzalez‐Rivas et al. 2020). This corroborates the findings of this study that heat stress reduces the carcass composition of the controlled broilers. However, the better carcass composition observed in heat‐stressed broilers on spirulina intervention suggests the potential of spirulina to enhance carcass development and cut‐part weights in broilers exposed to heat stress conditions. This observation may be connected to the ability of spirulina to improve FCR in heat‐stressed broilers, leading to increased ADG as observed in the current study. These findings align with Moustafa et al. (2021), who stated that the addition of 0.25, 0.5, and 1.0% spirulina to the diet of broilers raised under hot and thermoneutral conditions reduced abdominal fat content and improved carcass composition. These results also support the findings of Kaoud (2015) and Khan et al. (2020), who found an increased dressing percentage in broilers fed with 0.1% and 0.2% spirulina. This enhancement in carcass composition can be justified by the ability of spirulina to provide essential nutrients and improve intestinal morphology in heat‐stressed broilers, resulting in nutrient conversion to lean meat (Tavernari et al. 2018).

Heat‐stressed broilers on spirulina intervention had higher heart, liver, and gizzard weights than the control group, which could be related to higher metabolic activities of these organs to counterbalance the adverse effects of heat stress. The significantly higher heart, liver, and gizzard weights in heat‐stressed broilers following spirulina intervention could be attributed to hypertrophy, leading to an increase in the size of the organs. These observations contrasted with the earlier research by Abdel‐Moneim et al. (2022a) in heat‐stressed broilers fed a diet supplemented with spirulina at 0.5% and 1.0% for 42 days. Research has found reduced immune organ (spleen and thymus) weights in heat‐stressed broilers (Apalowo et al. 2024). The current study revealed that the relative weights of immune organs were higher in heat‐stressed broilers on spirulina intervention than in the control group, which agrees with the findings of Kaoud (2015) in broilers fed 0.15% spirulina.

Hematological indicators are used to ascertain the state of health of farm animals and assist in analyzing stress levels owing to a variety of stressors (Ogbuewu et al. 2015; Nassar et al. 2023). The significantly higher concentrations of Hb, RBC, and WBC in heat‐stressed broilers on spirulina intervention imply a superior ability of spirulina to support the production of these blood components, leading to improved weight gain. The improved Hb, RBC, and WBC values in heat‐stressed broilers support the earlier view of Attia et al. (2023) and Abdel‐Moneim et al. (2022a) that spirulina is rich in essential nutrients and important bioactive compounds that support blood formation. Aqueous extracts of spirulina has been reported to normalize the hematological profiles during an anemic state in animals other than broilers (Selmi et al. 2011). However, these results contradict the findings of Alwaleed et al. (2021), who found that supplementing the broiler diet with spirulina at 0.5 or 1.0% did not increase RBC count. The low concentrations of Hb, RBC, and WBC observed in heat‐stressed broilers that did not receive spirulina treatment are expected, given that heat stress has been shown to have negative impacts on the hematological profile of broilers (Apalowo et al. 2024). These findings posit that spirulina treatment can enhance the hematological profiles of heat‐stressed broilers.

Blood proteins (total proteins, albumin, and globulin) are excellent indicators of both the metabolism of ingested feeds and the state of body cells, tissues, and organs (Ogbuewu et al. 2015). The higher the level of blood proteins, the better the quality of protein present in the feed or feedstuff (Ahiwe et al. 2024; Son et al. 2024). The increased blood protein values in heat‐stressed broilers on spirulina intervention suggest that spirulina contains high‐quality protein. Serum globulin is essential in combating disease and has been found to enhance immune functions in animals (Kolluri et al. 2022). The significantly higher serum globulin values in heat‐stressed broilers on spirulina intervention could be connected to enhanced immune functions as spirulina increase antibody production in farm animals (Kolluri et al. 2022). A report by Song et al. (2018) revealed that heat‐stressed broilers had lower concentrations of IgA, IgM, and IgG, which agrees with the results of the controlled broilers. In the current study, spirulina increased the levels of IgM, IgG, and IgA in broilers exposed to heat stress, which confirmed the immuno‐modulatory property of spirulina as reported by Khan et al. (2020) and Moustafa et al. (2021).

Uric acid is a waste formed during protein breakdown in avian species. A positive correlation exists between poor protein quality and elevated serum uric acid levels in animals (Ogunwole et al. 2017). The decreased uric acid levels in heat‐stressed broilers as recorded in this meta‐analysis indicate the high quality of protein contained in spirulina. Creatinine, a waste product of muscle metabolism, was reduced in the serum of heat‐stressed broilers on spirulina intervention, implying that these birds were not meeting their glucose requirements from non‐carbohydrate sources such as amino acids and lactate. This finding is in line with Moustafa et al. (2021), who reported that the addition of spirulina in chicken diet at 0.5, 1.0, and 1.5% improved renal biomarkers in heat‐stressed broilers. Enemor et al. (2005) reported that AST and ALT leak from liver cells to the bloodstream when there is hepatocellular injury. The reduction in concentrations of serum AST and ALT in heat‐stressed broilers on spirulina intervention indicates enhanced renal function and liver enzyme activity. This trend supports previous reports on the hepatoprotective properties of spirulina in animals (Mazokopakis et al. 2014; Mohamed et al. 2021).

Hyperlipidemia, also known as abnormal fat levels in the blood, is one of the most frequent lipid metabolism disorders and is connected with low levels of total cholesterol, triglycerides, HDL, and elevated LDL levels. The current study revealed that spirulina intervention reduced the levels of total cholesterol, triglycerides, and LDL in the serum of heat‐stressed broilers. This suggests the ability of bioactive to stimulate the release of adenosine 5′‐monophosphate‐activated protein kinase (AMPK), specifically the alpha (α) unit, the regulating pathway that downregulates the fat synthesis‐related genes through the 3‐hydroxy‐3‐methyl glutaryl coenzyme A reductase (Li et al. 2018). Another likely explanation for the hypolipidemic effect of spirulina may be connected to the capacity of polyphenolic compounds contained in spirulina (Abdel‐Moneim et al. 2020b), such as acacetin, pinocembrin, ferulic acid, caffeic acid, and several others to inhibit cholesterol biosynthesis and uptake in the intestine (Deng and Chow 2010). The finding of this study agrees with the results of other authors (Mirzaie et al. 2018; Di Nicolantonio et al. 2020; Elbaz et al. 2022), who found hypolipidemic effects of dietary spirulina supplementation in animals. C‐phycocyanin (C‐PC), the novel pigment protein in spirulina known for its anti‐inflammatory, immune‐regulating, and antioxidant properties, is reported to have a high ability to bind to bile acids and excretion of cholesterol, suggesting the cholesterol‐lowering effects in stressed broilers (Nagaoka et al. 2005).

Broilers produce reactive oxygen species when exposed to excessive heat stress due to the accumulation of superoxide anions in the mitochondria, resulting in oxidative stress and, consequently, increasing MDA levels, an index of lipid peroxidation (broilers (Apalowo et al. 2024). Superoxide dismutase assists in converting superoxide anions into hydrogen peroxide (H_2_O_2_), whereas GPx catalyzes the breakdown of harmful H_2_O_2_ to water or the equivalent alcohols (Park et al. 2018; Abdel‐Daim et al. 2020). A growing body of evidence indicates that oxidative stress impairs body metabolic processes and growth in chickens (Liu et al. 2020; Elbaz et al. 2022). Research has demonstrated that heat stress causes redox imbalance by reducing the concentrations of SOD and GPx and increasing MDA concentrations (Elbaz et al. 2022; Apalowo et al. 2024). However, spirulina treatment due to its rich antioxidant components (i.e., selenium, phenolic compounds, ascorbic acid, C‐PC, α‐tocopherol, and β‐carotene) restored the negatively disturbed redox balance linked to heat stress in broilers by increasing the concentrations of SOD and GPx and decreasing the MDA values (Farag et al. 2016; Pestana et al. 2020). The decreased MDA in the current meta‐analysis suggests that the cells are not under oxidative stress. Studies showed that spirulina administration increased the levels of SOD and GPx while reducing MDA levels in broilers (Mirzaie et al. 2018; Abdel‐Moneim et al. 2022a), which agrees with the findings of this meta‐analysis.

## 5 Conclusion and Future Research Direction

The present meta‐analysis showed that spirulina intervention enhanced growth performance, organ weights, carcass composition, blood characteristics, antioxidative status, and immune functions of broilers exposed to heat stress conditions, contrary to the findings of other researchers. The enhanced production traits and health status of heat‐stressed broilers on spirulina intervention, as identified in this meta‐analysis, emphasize the need to develop policies that promote the use of spirulina to mitigate adverse impacts of heat stress on broiler productivity. The analysis also revealed superior ADG in heat‐stressed broilers that received ≤ 1.0% spirulina than those that received > 1.0% spirulina. However, the best dose of spirulina that optimize growth performance parameters of heat‐stressed broilers should be determined using a quadratic optimization model to avoid the wastage of spirulina additives. There is scanty publications on the effect of spirulina extracts on productivity of broilers exposed to heat stress conditions and future research should channeled to this area. This study also suggested that the analyzed moderators did not explain the sources of heterogeneity variance among the studies used for meta‐analysis. This indicates that more research is needed to identify other moderators that are responsible for the heterogeneity variance. Interestingly, this study has set guidelines for standardized experimental designs on the potential of spirulina intervention in mitigating the adverse consequences of heat stress in broiler performance in the future.

## Author Contributions

Christian Anayo Mbajiorgu and Ifeanyichukwu Princewill Ogbuewu: Conceptualized and designed the study. Christian Anayo Mbajiorgu and Ifeanyichukwu Princewill Ogbuewu: Designed the search strategy, and performed the literature search, data extraction, and data analysis. Christian Anayo Mbajiorgu: Produced the draft. Ifeanyichukwu Princewill Ogbuewu: Review and edit the draft. The authors read and approved the final draft.

## Ethics Statement

The authors adhered to the ethical policies of the journal, as noted on the journal's author guidelines page. This study is a meta‐analysis and does not need ethics approval.

## Conflicts of Interest

The authors declare no conflicts of interest.

## Data Availability

The data that support the findings of this study are available from the corresponding author upon reasonable request.
